# Detection of Shiga Toxin-Producing *Escherichia coli* (STEC) in the Endocervix of Asymptomatic Pregnant Women. Can STEC Be a Risk Factor for Adverse Pregnancy Outcomes?

**DOI:** 10.3389/fendo.2022.945736

**Published:** 2022-07-20

**Authors:** María Luján Scalise, Nicolás Garimano, Marcelo Sanz, Nora Lia Padola, Patricia Leonino, Adriana Pereyra, Roberto Casale, María Marta Amaral, Flavia Sacerdoti, Cristina Ibarra

**Affiliations:** ^1^ Laboratorio de Fisiopatogenia, Instituto de Fisiología y Biofísica, IFIBIO-Houssay (UBA-CONICET), Departamento de Ciencias Fisiológicas, Facultad de Medicina, Universidad de Buenos Aires, Buenos Aires, Argentina; ^2^ CIVETAN-Centro de Investigación Veterinaria Tandil (CONICET, CICPBA), Facultad de Ciencias Veterinarias, Tandil, Argentina; ^3^ Departamento de Obstetricia, Hospital Nacional “Prof. A. Posadas”, Buenos Aires, Argentina

**Keywords:** Endocervical microbiota, pregnancy, STEC, virulence factors, Stx2

## Abstract

The presence of *Escherichia coli* in the vaginal microbiome has been associated with pregnancy complications. In previous works, we demonstrated that Shiga toxin-producing *Escherichia coli* (STEC) can produce abortion and premature delivery in rats and that Shiga toxin type 2 (Stx2) can impair human trophoblast cell lines. The hypothesis of this work was that STEC may colonize the lower female reproductive tract and be responsible for adverse pregnancy outcomes. Thus, the aim of this work was to evaluate the presence and prevalence of virulence factor genes from STEC in the endocervix of asymptomatic pregnant women. For that purpose, endocervical swabs were collected from pregnant women during their prenatal examination. Swab samples were enriched in a differential medium to select *Enterobacteria*. Then, positive samples were analyzed by PCR to detect genes characteristic of *Escherichia* sp. (such as *uidA* and *yaiO*), genes specific for portions of the *rfb* (O-antigen-encoding) regions of STEC O157 (*rfb*_O157_), and STEC virulence factor genes (such as *stx1*, *stx2*, *eae*, *lpfA*_O113_, *hcpA*, *iha*, *sab, subAB*). The cytotoxic effects of stx2-positive supernatants from *E. coli* recovered from the endocervix were evaluated in Vero cells. Our results showed that 11.7% of the endocervical samples were positive for *E. coli*. Additionally, we found samples positive for *stx2* and other virulence factors for STEC. The bacterial supernatant from an isolate identified as *E. coli* O113:NT, carrying the *stx2* gene, exhibited cytotoxic activity in Vero, Swan 71 and Hela cells. Our results open a new perspective regarding the presence of STEC during pregnancy.

## Introduction

Shiga toxin-producing *Escherichia coli* (STEC), a bacterium that belongs to the family *Enterobacteriaceae*, can cause severe foodborne diseases. The main reservoir for STEC is the intestinal tract of cattle, and the bacterium can survive for months in soil, water, or organic material (1). The main route of transmission is through contaminated food, mainly minced meat (undercooked meat below 71°C), unpasteurized food, contaminated vegetables and person-to-person contact by the fecal-oral route (2, 3).

STEC comprises strains producing two Shiga toxins: Stx type 1 (Stx1) and Stx type 2 (Stx2) (4) and its variants, Stx1 (a – c) and Stx2 (a – k) (5–7). Not all Stx subtypes have been associated with severe illness (8). In this sense, it has been described that Stx2a and Stx2c are clinically more related with severe cases of hemolytic uremic syndrome (HUS), and that STEC O157 strains carrying stx2a predominate in human infections, causing more severe disease symptoms than those carrying stx2c (9, 10).

Stx is the main virulence factor of STEC and, in combination with other virulence factors, contributes to the pathogenic potential of STEC strains. In this sense, intimin (*eae* gene) plays an essential role in the intimate attachment and colonization of intestinal cells of STEC O157:H7, although many STEC isolates lacking the *eae* gene are also able to efficiently colonize the human gut (11). Many other proteins, such as the long polar fimbriae (Lpf) (12), the hemorrhagic *coli* pilus (Hcp) (13), the IrgA homolog adhesin (IhA) (14), and the STEC autotransporter contributing to biofilm formation (Sab) (15), are also involved in the adherence of O157 and non-O157 STEC strains. Non-O157 STEC strains also produce Subtilase cytotoxin (SubAB), able to cause cytotoxic effects in epithelial cells (16).

It is currently known that *E. coli* strains can colonize the vagina, usually asymptomatically, although epidemiological studies have shown that the presence of these bacteria in the female reproductive tract may be a risk factor for pregnancy (17). Some studies developed in Argentina and other countries have shown a high prevalence of *E. coli* in vaginal microbiota of adult women (17, 18). However, to our knowledge, there are no reports about the effects of STEC infection on human pregnancy, although some reports have indicated that STEC transmission from the mother to the child during delivery can cause neonatal HUS (19) or STEC-mediated HUS during pregnancy (20). In Argentina, the most common STEC serotype is O157:H7 (21, 22). Nevertheless, the appearance of non-O157 STEC strains is a clear evidence of the dynamic genome of these *E. coli* pathogens and their ability to transfer or acquire important virulence factors (23, 24). Since there are no epidemiological studies regarding STEC infections during pregnancy, it is difficult to know their impact on reproductive health.

Previous works in our laboratory have demonstrated that Stx2 injection in pregnant rats in early and late pregnancy causes premature delivery, miscarriage and impairments in placental development (25, 26). Also, we have shown that the immunization of rats against Stx2 can prevent the detrimental effects of the toxin during pregnancy (27). Taking these previous findings into account, we hypothesized that STEC can colonize the endocervix and may be responsible for complications in pregnancy. The main goal of this work was to evaluate the presence and prevalence of virulence factor genes from STEC in the endocervix from asymptomatic pregnant women and to better understand the possible clinical relevance of this pathogen during human pregnancy.

## Materials and Methods

### Endocervical Samples

Asymptomatic pregnant women (from 17 to 37 years old) with gestational age from 12 to 34 weeks were enrolled (n=103) during their prenatal examination in the Obstetrics Service of the Maternal and Child Department of the Prof. A. Posadas National Hospital (Buenos Aires, Argentina) between January and March 2019. Endocervical swab samples were collected under direct visualization during a speculum examination. Swabs were transported in sterile tubes containing Cary Blair transport medium (Britania, Argentina) and stored at 4-8°C until used.

### Selection of *Enterobacteria* From Endocervical Samples

Endocervical swabs were enriched in 3 mL of Tryptic Soy Broth (Oxoid, UK) overnight (ON) at 37°C and shaken at 150 rpm. Then, cultures were streaked into Sorbitol MacConkey (SMAC) agar (Oxoid, UK) and incubated ON at 37°C. SMAC agar was used for a preliminary selection of *Enterobacteria* based on a differential sorbitol and lactose fermentation profile. SMAC agar is recommended as a selective and differential medium for the detection of STEC O157:H7. *Enterobacteria* capable of fermenting sorbitol form pink colonies on SMAC agar, while non-sorbitol fermenting bacteria, such as STEC O157:H7, form white/colorless colonies.

### Detection of *E. Coli* and STEC O157:H7 in Enriched Endocervical Samples by PCR

Total genomic DNA from colonies grown in SMAC agar was obtained to detect the presence of *E. coli* by PCR. DNA was purified using PURO bacteria Kit (PB-L products, Bio-Logicos, Argentina) according to the manufacturer’s instructions and quantified by Nanodrop One (Thermo Fisher Scientific, USA). Specific genes encoding for the enzyme beta-glucuronidase (*uidA* gene) (28) and external membrane protein of *E. coli* (*yaiO* gene) (29), and genes specific for portions of the *rfb* (O-antigen-encoding) regions of STEC O157 (*rfbO_157_*gene) (11) were detected by PCR using specific primers (**Table 1**).

**Table 1:** Primers used.

Gene	primers	Sequence (5´-3´)	Amplicon size (bp)
*uidA*	*uidA for* *uidA rev*	*TGGTAATTACCGACGAAAACGGC* *ACGCGTGGTTACAGTCTTGCG*	162
*yaiO*	*yaiO for* *yaiO rev*	*TGATTTCCGTGCGTCTGAATG* *ATGCTGCCGTAGCGTGTTTC*	116
*rfbO_157_*	*rfbO_157_ for* *rfbO_157_ rev*	*CGGACATCCATGTGATATGG* *TTGCCTATGTACAGCTAATCC*	259

PCRs were performed in a total volume of 20 µL: 6 µL H_2_O, 3 µL DNA, 0.5 µL of each forward and reverse primer (10 µM) and 10 µL Master Mix 2X Kit (M024, Inbio Highway, Argentina). The thermocycler (Thermo Fisher Scientific, USA) program used consisted of one cycle at 95°C for 10 min followed by 35 cycles of 94°C for 40 s, 58°C for 30 s, and 72°C for 60 s, ending with one cycle of 72°C for 2 min. A total of 10 μL of each PCR reaction product was loaded into an agarose gel to confirm the presence and size of the corresponding amplicon.

Samples positive for *yaiO* were analyzed for *rfbO157* as well as for the presence of other STEC virulence factor genes, including: *stx1*, *stx2*, *stx2a*, *stx2c* (4), *subAB* (30), *eae* (31), *lpfAO_113_* (12), *hcp* (13), *iha* (32) and *sab* (15) by PCR (**Table 2**).

**Table 2:** Evaluation of STEC virulence factors, primer sequences and size of amplicons .

Virulence factor	primers	Sequence (5´-3´)	Amplicon size (bp)
**Shiga toxin** **type 1**	*stx1 for* *stx1 rev*	*ATGTCATTCGCTCTGCAATAGGTAC* *GAAGAAGAGACTGAAGATTCCATCTG*	1020
**Shiga toxin** **type 2**	*stx2 for* *stx2 rev*	*GGCACTGTCTGAAACTGCTCCTGT* *ATTAAACTGCACTTCAGCAAATCC*	627
**Shiga toxin** **type 2a**	*stx2a for* *stx2a rev*	*GCGATACTGRGBACTGTGGCC* *CCGKCAACCTTCACTGTAAATGTG*	349
**Shiga toxin** **type 2c**	*stx2c for* *stx2c rev*	*GAAAGTCACAGTTTTTATATACAACGGGTA* *CCGGCCACYTTTACTGTGAATGTA*	177
**Subtilase**	*subAB for* *subAB rev*	*TATGGCTTCCCTCATTGCC* *TATAGCTGTTGCTTCTGACG*	556
**Intimin**	*eae for* *eae rev*	*GGAACGGCAGAGGTTAATCTGCAG* *GGCGCTCATCATAGTCTTTC*	346
**Long polar** **fimbriae A_O113_**	*lpfA_O113_ for* *lpfA_O113_ rev*	*ATGAAGCGTAATATTATAG* *TTATTTCTTATATTCGAC*	573
**Hemorragic *coli* pilus**	*hcp for* *hcp rev*	*TCGCTAGTTGCTGACAGATTT* *AATGTCTGTTGTGTGCGACTG*	868
**IhA adhesion**	*iha for* *iha rev*	*CAGTTCAGTTTCGCATTCACC* *GTATGGCTCTGATGCGATG*	1305
**Sab (STEC AT) mediating biofilm formation**	*sab for* *sab rev*	*GGTGGATACAGCAGGTAATG* *TATCTCACCACCTGCTATCG*	163

### Cell Line Cultures

The Vero cell line (Vero), derived from African green monkey kidney, was purchased from the American Type Culture Collection (ATCC-CCL-81, Manassas, VA, USA). The HeLa cell line (ATCC-CCL-2), derived from a human cervix adenocarcinoma, was used as a human endocervical model. The Swan 71 cell line, derived by telomerase-mediated transformation of a 7-week cytotrophoblast isolate, was kindly provided by Dr. Gil Mor, Yale University, New Haven, CT, USA (33).

All cell lines were cultured in Dulbecco’s Modified Eagle Medium/Nutrient Mixture F-12 (DMEM-F12 medium, Sigma Aldrich, USA) supplemented with 10% Fetal Bovine Serum (FBS, Internegocios S.A., Argentina), 100 U/mL penicillin/streptomycin, and 2 mM L-glutamine (GIBCO, USA), and grown at 37°C in a humidified 5% CO_2_ incubator. For growth-arrested conditions, the medium was used without FBS.

### Evaluation of the Cytotoxic Effects of Bacterial Supernatants From Bacteria Isolated From the Endocervical Samples

Endocervical samples that were positive for *stx2* by PCR were grown ON in Luria-Bertani Broth (LB) (Sigma Aldrich, USA) at 37°C with shaking at 150 rpm. Then, the cultures were diluted (1:20) in DMEM-F12 medium (Sigma Aldrich, USA), supplemented with 1 mM of HEPES buffer solution (4-(2-Hydroxyethyl)piperazine-1-ethanesulfonic acid, N-(2-Hydroxyethyl)piperazine-N′-(2-ethanesulfonic acid)) (GIBCO, USA) and grown until exponential phase (OD_600_ = 0.4-0.5) at 37°C with shaking at 150 rpm. Subsequently, bacterial supernatants were collected after centrifugation at 10,000 x *g* for 5 min and filtered (0.22-μm pore-size filter; Millipore, USA). In some experiments, to induce Stx2 production, mitomycin-C (1 µg/mL) was added at the exponential phase and the bacterial culture was grown for additional 3 h (34).

### Cell Viability Assays

Cell viability assays were performed as previously described (35). Briefly, 15x 10^3^ cells/well (Vero, Swan 71, and HeLa) were seeded in 96-well plates and grown at 80% of confluence. Then, cells were exposed for 72 h to different concentrations (1x10^-6^ – 0.1 µg/mL) of purified Stx2 (Phoenix Laboratory, Tufts Medical Center, Boston, USA) or to serial dilutions of bacterial supernatant (10^-1^-10^-6^) in growth-arrested conditions. After treatment, cells were incubated with a neutral red solution (50 µg/mL) at 37°C for 1 h in 5% CO_2_. Then, cells were washed and fixed with 1% CaCl_2_-1% formaldehyde and finally lysed with 1% acetic acid in 50% ethanol solution to solubilize the neutral red uptake by cells. Absorbance in each well was read at 540 nm in an automated plate spectrophotometer (RT-6000, Rayto Life and Analytical Sciences Co. Ltd., China). Results are expressed as percentage of cell viability, where 100% represents control cells without toxin or supernatant treatment. The 50% cytotoxic dose (CD_50_) of Stx2 and the bacterial supernatant corresponded to the dilution required to kill 50% of cells.

### Stx2-Neutralization Assay

For the neutralization assay, bacterial supernatant in a dilution required to kill 50% of Vero cells (CD_50_) was co-incubated with 25 µg/mL of the mouse monoclonal antibody 2E11 against the A-subunit of Stx2 (anti-Stx2 mAb) at 37°C for 1 h (36). The mixture was added to a 96-well culture plate containing Vero cells grown at 80% of confluence and then incubated for 72 h. The cytotoxicity was analyzed by neutral red uptake as previously described. The CD_50_ of purified Stx2 (1 ng/mL) was used as control.

### Isolation and Characterization of STEC Samples From the Endocervix

The enriched bacterial culture from endocervical samples carrying the *stx2* gene and showing cytotoxic effects on Vero was streaked onto LB agar plates and incubated ON at 37°C. Grown colonies were picked and added in 96-well plates and grown in LB medium ON at 37°C with agitation at 150 rpm. The next day, wells of each column were pooled and the DNA was purified to test the presence of the *stx2* gene. Afterward, individual colonies grown in each well of stx2-positive columns were directly picked and confirmed to be STEC by testing the presence of the *stx2* gene by PCR and by Vero cell assays. Isolates were then sub-cultured for further genetic or phenotypic typing.

### Determination of the Serotypes of STEC Isolates

The serotypes (O and H) of STEC isolates were determined by microagglutination, as described by Blanco etal. (37).

### Ethical Approval

This study had the approval of the Human Research Ethics Committee of the Prof. A. Posadas National Hospital, Buenos Aires, Argentina (Ref # 423 EMnPeSe/20), in accordance with the Argentine Good Clinical Practice Guidelines. All pregnant women were thoroughly informed about the purpose of the study and provided a written informed consent. The exclusion criteria were: women with genital papilloma virus infection (HPV), human immunodeficiency virus (HIV), pelvic inflammatory disease (PID) and sexually transmitted diseases such as chlamydia, gonorrhea, and genital herpes.

### Statistical Analysis

Data were plotted and statistically analyzed using Graph Pad Prism 5.0 (San Diego, CA, USA). Cytotoxicity curves were fitted using a four-parameter logarithmic regression. Statistical significance for all experiments was assessed using analysis of variance (ANOVA) with Tukey’s multiple comparison test as a *posteriori* test. In all cases, statistical significance was set at *p < 0.05.

## Results

### Detection and Identification of *E. coli* From Endocervical Swab Samples

Sixteen out of the 103 endocervical swab samples developed colonies on SMAC agar. Only non-sorbitol fermenting colonies were observed. Fifteen out of those sixteen samples had the *uidA* gene and 12 out of the 16 had the *yaiO* gene (**Table 3**). Considering that the *yaiO* gene is more specific than the *uidA* gene for the identification of *E. coli*, only those containing the *yaiO* gene were considered positive for *E. coli* (29). One sample (S12) was negative for both genes (**Table 3**). Therefore, 12 out of the 103 endocervical samples recruited for this study (11.7%) were considered positive for *E. coli* spp. in the endocervical microbiota. The absence of sorbitol-fermenting colonies (characteristic of O157:H7 *E. coli*) on SMAC agar was confirmed by PCR (none of the samples evaluated were positive for the *rfb_O157_* gene) (**Table 3**).

**Table 3:** Genotypic characterization of *E. coli* in endocervical samples grown on SMAC agar.

Endocervical samples SMAC agar POS	*uidA*	*yaiO*	*rfbO_157_*
**S1**	POS	POS	–
**S2**	POS	POS	–
**S3**	POS	POS	–
**S4**	POS	POS	–
**S5**	POS	–	–
**S6**	POS	POS	–
**S7**	POS	–	–
**S8**	POS	POS	–
**S9**	POS	POS	–
**S10**	POS	–	–
**S11**	POS	POS	–
**S12**	–	–	–
**S13**	POS	POS	–
**S14**	POS	POS	–
**S15**	POS	POS	–
**S16**	POS	POS	–

(-): NEGATIVE (POS): POSITIVE

### Detection of STEC Virulence Genes in Bacterial Isolates Obtained From Endocervical Swab Samples

The presence of STEC virulence factors was analyzed in the 12 endocervical samples that developed colonies on SMAC agar and were confirmed positive for *E. coli* by the presence of the *yaiO* gene. Seven out of the 12 samples (58.3%) amplified for the *stx2* gene, 5 out of the 12 (41.7%) for the *lpf_AO113_* gene, 8 out of the 12 (66.7%) for the *hcpA* gene and 3 out of the 12 (25%) for the *iha* gene (**Table 4**). None of them were positive for the *eae*, *stx1* or *sab* genes.

**Table 4:** Genotypic identification of STEC virulence factors in *E. coli*-positive samples .

Endocervical sample *E. coli* POS	*stx1*	*stx2*	*subAB*	*eae*	*lpfA_0113_*	*hcpA*	*iha*	*sab*
**S1**	–	–	–	–	POS	–	–	–
**S2**	–	–	–	–	POS	POS	–	–
**S3**	–	–	–	–	POS	POS	–	–
**S4**	–	POS	–	–	–	POS	–	–
**S6**	–	POS	–	–	POS	POS	POS	–
**S8**	–	POS	–	–	–	–	–	–
**S9**	–	POS	–	–	–	POS	–	–
**S11**	–	POS	–	–	–	POS	POS	–
**S13**	–	POS	–	–	–			–
**S14**	–	POS	–	–	POS	POS	POS	–
**S15**	–	–	–	–	–	–	–	–
**S16**	–	–	–	–	–	POS	–	–

(-): NEGATIVE: (POS): POSITIVE

### Evaluation of the Cytotoxic Activity of Bacterial Supernatants Obtained From Endocervical Swab Samples Positive for the *Stx2* Gene

**Figure 1:**
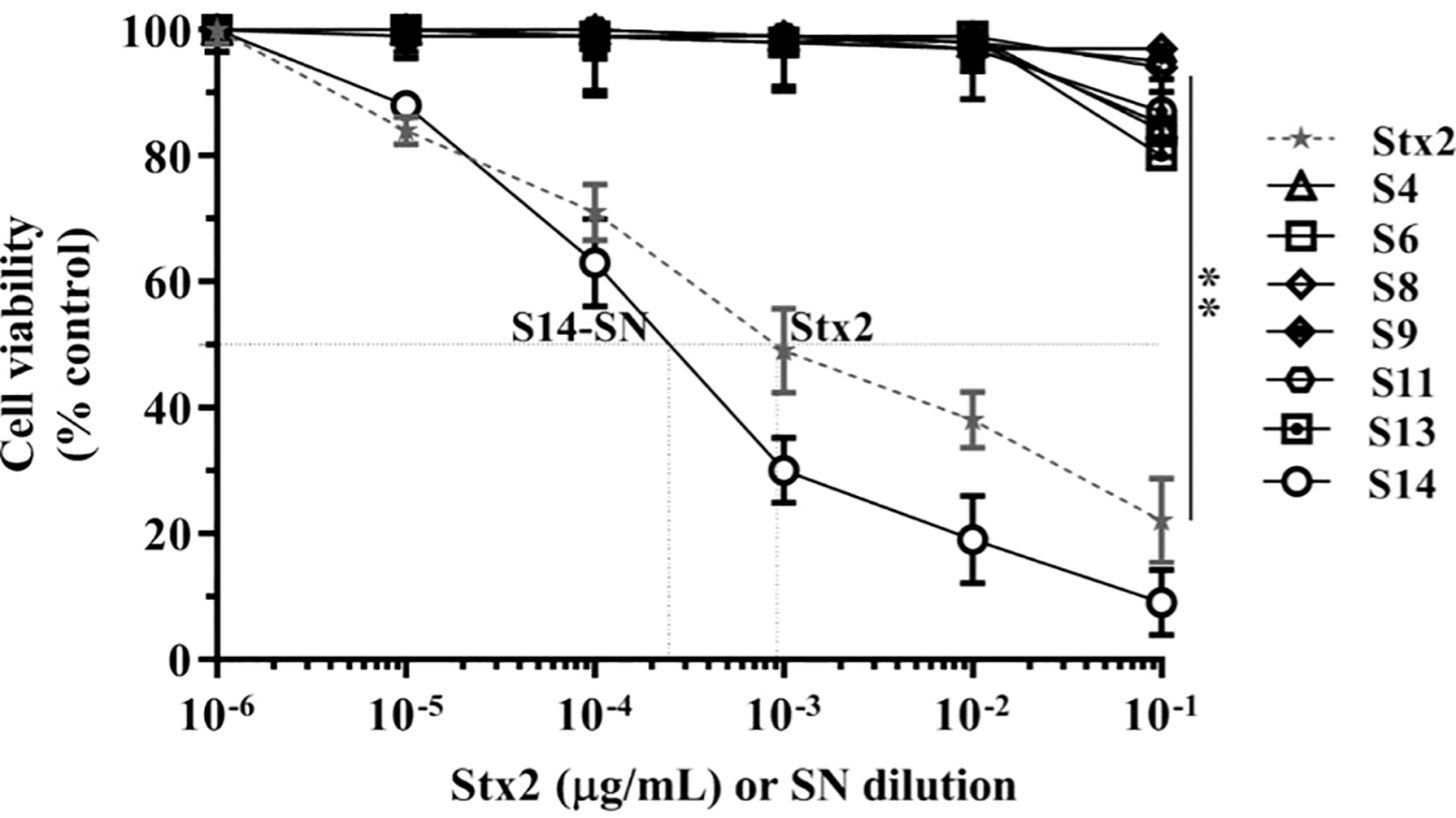
Cytotoxicity of *E. coli*-positive endocervical samples carrying the *stx2* gene on the viability of Vero cells. Vero cells were exposed to serial dilutions of bacterial supernatants (SN) from endocervical samples carrying the *stx2* gene or different concentrations of purified Stx2 under growth-arrested conditions. Cell viability was determined by neutral red uptake after 72 h of incubation, and 100% represents cells incubated under identical conditions but without treatment. Data are shown as means ± S.D from at least three independent experiments performed in triplicate. **p < 0.01.

The cytotoxic activity of filter-sterilized bacterial supernatants obtained from endocervical samples positive for *E. coli* carrying the *stx2* gene was evaluated in Vero cells (**Figure 1**).

Incubation of Vero cells for 72 h with serial dilutions of the bacterial supernatant corresponding to sample 14 (S14-SN) caused a significant cytotoxicity in a dose-dependent manner, reaching a CD_50_ at 2x10^-4^ dilution. The CD_50_ of Stx2 in S14-SN was equivalent to that elicited by approximately 5 µg/mL of purified Stx2 (**Figure 1**). In contrast, the other filter-sterilized bacterial supernatants showed no cytotoxic effects on the viability of Vero cells, even if previously grown with mitomycin-C, an inductor of *stx2* phages (data not shown).

### Neutralization of Stx2 Cytotoxicity of Bacterial Supernatants From Endocervical Samples

**Figure 2:**
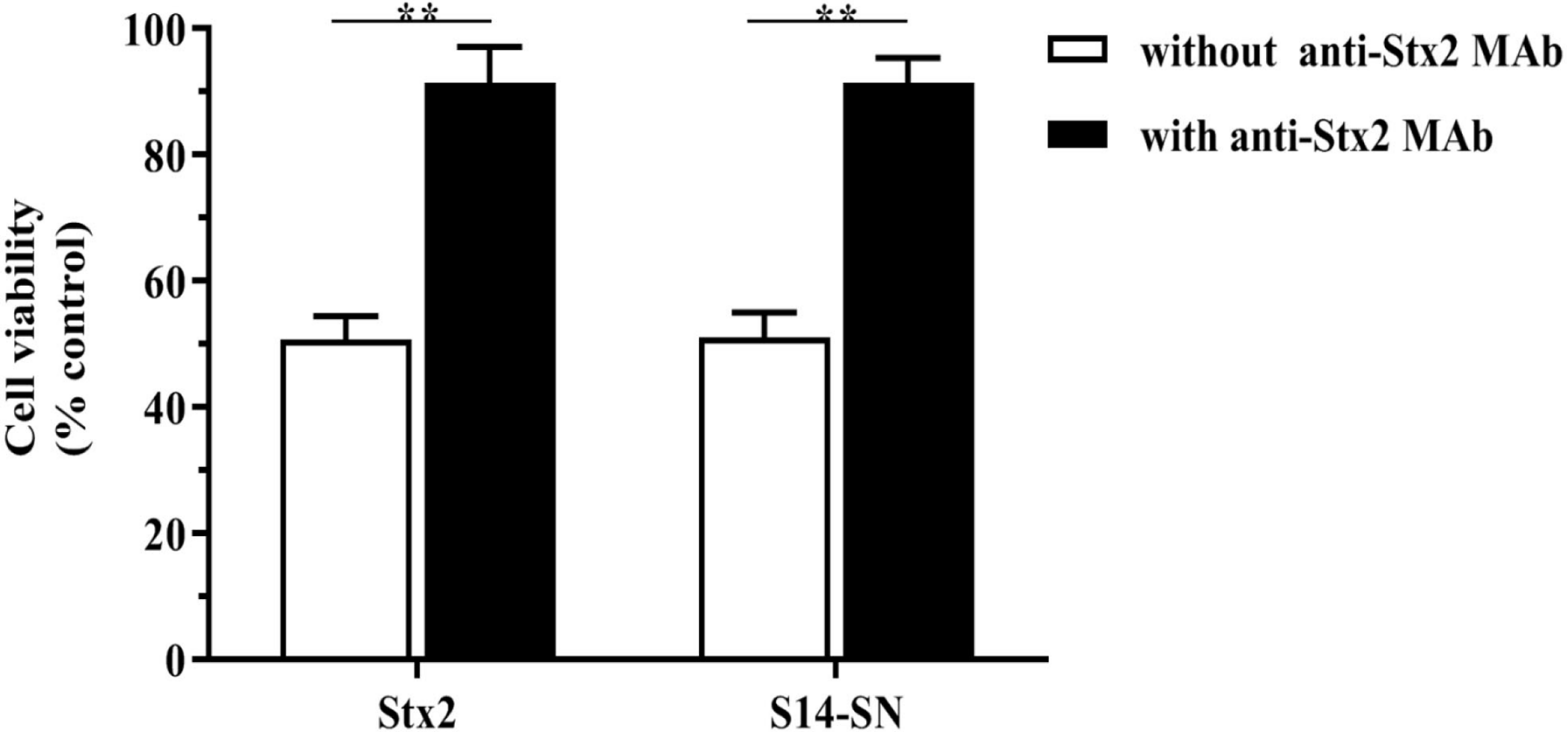
Neutralization of the Stx2 cytotoxicity of bacterial supernatants isolated from endocervical samples on the viability of Vero cells. The bacterial supernatant from the endocervical sample 14 (S14-SN, dilution 1:5000) was preincubated with the mouse monoclonal antibody 2E11 against the A-subunit of Stx2 (anti-Stx2 mAb, 25 µg/mL) at 37°C for 1 h. The mixture was then added to a 96-well culture plate containing Vero cells and incubated for 72 h. The CD_50_ of purified Stx2 (1 ng/mL) incubated with anti-Stx2 mAb was used as control. Cell viability was analyzed by neutral red uptake. Data are shown as mean ± S.D from at least three independent experiments performed in triplicate. **p < 0.01, n=3.

Neutralization studies were used to confirm that the significant decrease in Vero cell viability caused by S14-SN was due to the cytotoxic action of Stx2. The results showed that S14-SN cytotoxicity was prevented by preincubation of the bacterial S14-SN (1:5000) with the anti-Stx2 mAb (25 µg/mL) at 37°C for 1 h. Neutralization of purified Stx2 was used as control (**Figure 2**).

### Cytotoxic Effects of STEC-Positive Endocervical Samples on the Viability of Human Endocervical and Extravillous Trophoblast Cells

**Figure 3:**
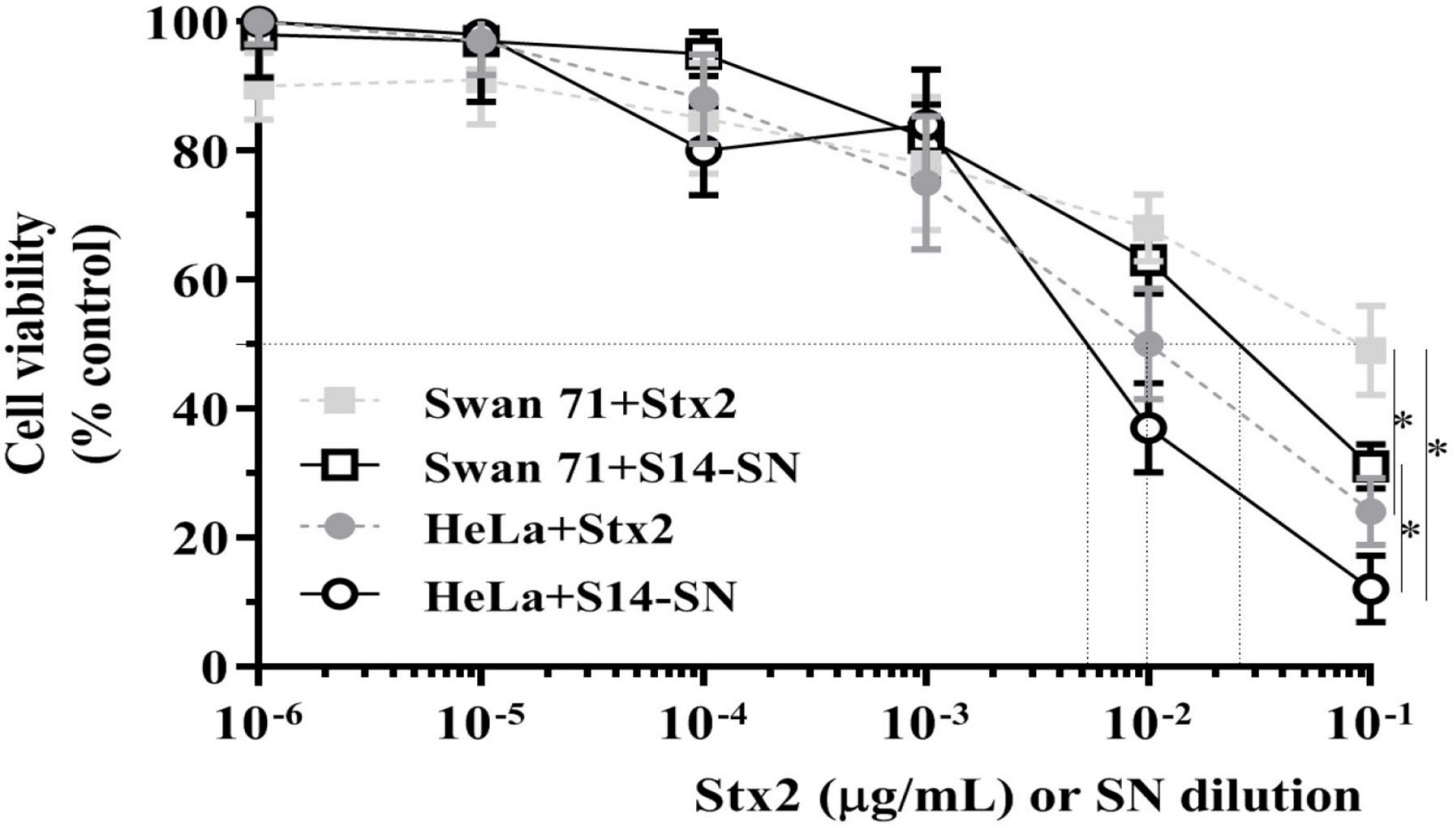
Cytotoxic effects of STEC-positive endocervical samples on the viability of Swan 71 and HeLa cell lines. Cells were exposed to purified Stx2 or serial dilutions of the supernatant from endocervical sample S14 (S14-SN) for 72 h. Cell viability was analyzed by neutral red uptake. Data are shown as mean ± S.D from at least three independent experiments performed in triplicate. *p < 0.05, n=3.

A significant cytotoxic effect was observed when monolayers of human endocervical (HeLa) cells were exposed to different concentrations of Stx2 or serial dilutions of the filter-sterilized bacterial supernatants or bacteria isolated from endocervical sample S14 (S14-SN) for 72 h. The CD_50_ was obtained with 10 ng/mL of purified Stx2 and with a 5x10^-3^ dilution of S14-SN. In agreement with the results described above, cytotoxicity was also observed with purified Stx2 on monolayers of extravillous trophoblasts (Swan 71 cells). The CD_50_ after 72 h of incubation with purified Stx2 was obtained at 100 ng/mL and with a dilution of approximately 2x10^-2^ of S14-SN (**Figure 3**). These results indicate that filter-sterilized bacterial supernatants from the STEC-positive endocervical microbiota can impair endocervical and trophoblast cell viabilities mediated by Stx2.

### Genotypic Characterization of STEC Isolated From an Endocervical Sample

**Figure 4:**
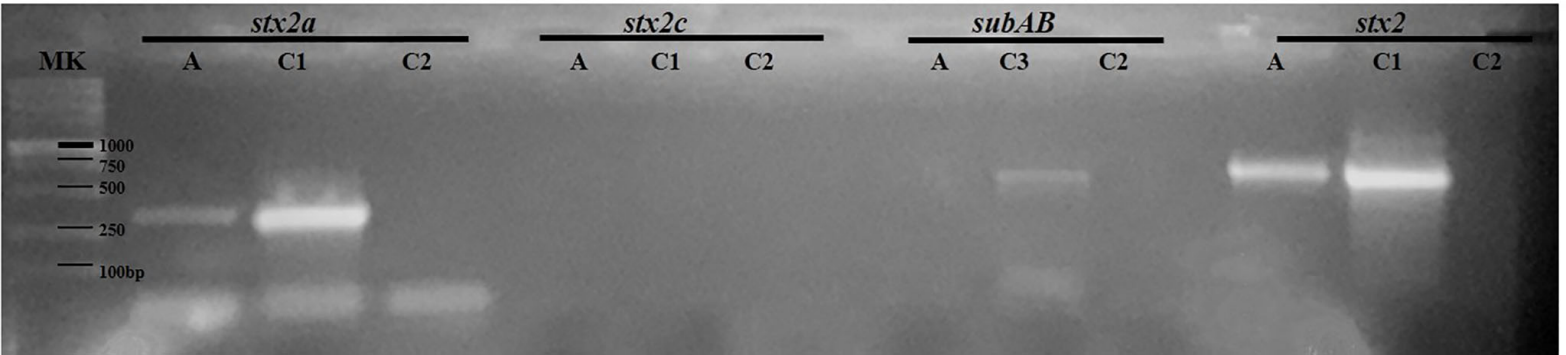
Characterization of a STEC strain isolated from an endocervical sample by PCR assays. The STEC strain named 123/21 was subtyped for the stx2 gene. The amplicons were seeded on 1.5% agarose gel for 30 min at 80 Volts. A: STEC strain 123/21. C1: *E. coli* O157:H7 strain 125/99 (stx2a+ 349 bp; stx2 627 bp), C2: negative control of PCR; C3: *E. coli* O113:H21 (subAB; 556 bp); DNA size marker (MK).

The bacterial isolate from endocervical sample S14, named STEC 123/21, was subtyped for *stx2* by PCR, using specific primers for the *stx2a* and *stx2c* genes (**Table 2**). **Figure 4** shows a representative gel of the STEC isolate subjected to PCR assay. Results showed PCR products of the expected sizes, consistent with the presence of the *stx2a* gene (**Figure 4**) and the absence of the *subAB* gene (S14 in **Table 3**).

### Serotypification of STEC Strain 123/21

**Figure 5:**
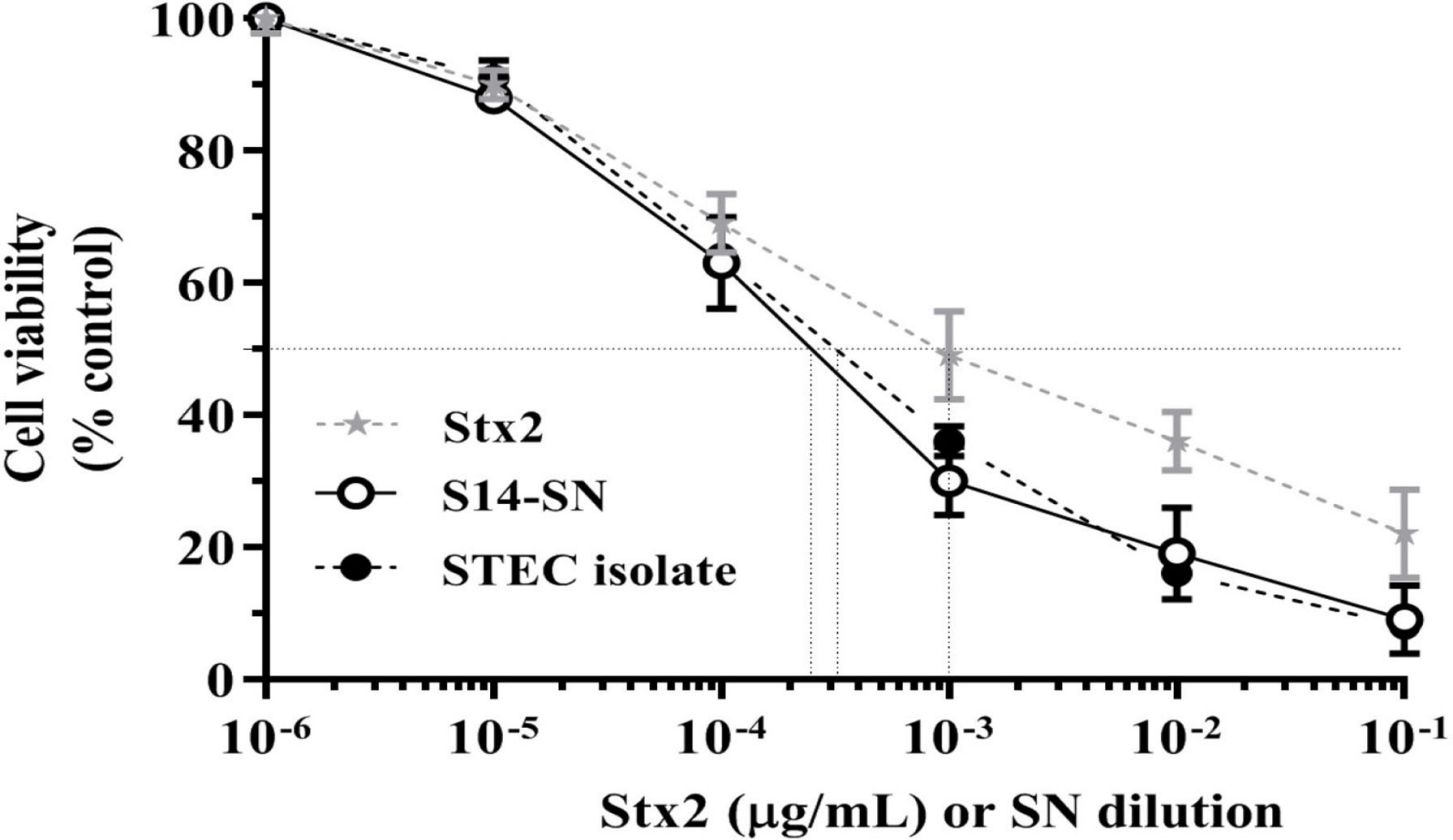
Cytotoxic effects of the STEC isolate O113:NT obtained from an endocervical sample on the viability of Vero cells. Cells were exposed to purified Stx2 or serial dilutions of supernatants from STEC O113:NT or sample S14 for 72 h. Cell viability was analyzed by neutral red uptake. Data are shown as mean ± S.D from at least three independent experiments performed in triplicate. *p < 0.05.

STEC strain 123/21 belonged to the O113 type, but the H type could not be determined (nondetermined, NT). The analysis of the supernatant from STEC O113:NT demonstrated that it was able to induce a marked decrease in cell viability. The cytotoxicity of the supernatant of this STEC isolate was similar to that obtained from the initial endocervical sample S14 containing other bacteria (SN-S14) (**Figure 5**).

## Discussion

The vaginal microbiota is a complex ecosystem consisting of around 200 species of microorganisms, being *Lactobacillus crispatus*, *L. jensenii* and *L. gasseri* the most represented species. Several studies have shown that around 70% of the vaginal microbiota in pregnant and non-pregnant women is composed of *Lactobacillus* (38–40). The substitution of *Lactobacillus* by pathogenic or opportunistic microorganisms is associated with the development of bacterial vaginosis and lower urinary tract infections, and the presence of non-*Lactobacillus* species has been associated with poor reproductive health and/or complications in pregnancy (41–44). In this regard, the presence of *E. coli* in the female reproductive tract has been found to be a risk factor for the progression of pregnancy (17, 45, 47).

The results of this work demonstrate the prevalence of *E. coli* in the endocervical microbiota in 11.7% of asymptomatic pregnant women. Previous studies performed in Argentina (18) and other countries of South America, Europe and North America (17) have reported an incidence similar to that found in this work. *E. coli* are commensal bacteria of the intestine of humans and animals, and some pathogenic strains, such as STEC, can cause moderate to severe gastrointestinal disease in humans. However, the risk of spontaneous abortion or preterm delivery in humans associated with STEC infection or its main virulence factor, Stx2, has not yet been evaluated.

Our results showed that 7 out of the 12 endocervical samples positive for *E. coli* carried the *stx2* gene, although only one of them (1/12, 8.3%) caused significant cytotoxicity in Vero cells due to the presence of Stx2 in the bacterial culture supernatant. The isolated STEC was identified as O113:NT Stx2a-positive. The Stx2 cytotoxicity evaluated by neutral red uptake method in the remaining stx2-positive *E. coli* samples was undetectable. We hypothesized that the composition of the endocervical gram-negative microbiota may be negatively regulating Stx2 expression and/or STEC growth, as previously described (46, 47). Moreover, the relationship between induction of Stx2-encoding phages and toxin production in STEC (48) may vary considerably in response to the microenvironment (49).

An interesting finding was the detection of *lpfA_0113_* and *hcpA* in our samples, considering that *lpf_AO113_*-positive STEC strains are associated with the appearance of small outbreaks of intestinal and extra-intestinal diseases in humans (50) and that the *hcpA* gene is associated with the pathogenicity of non-O157 STEC (51).

The fact that the microbiological selection by SMAC was negative for O157:H7, together with the absence of amplification for the *eae* gene that encodes for *Locus of Enterocyte Effacement* (LEE), led us to study the expression of the *iha* gene, which encodes an outer membrane adhesin protein. This adhesin was detected in 25% (3/12) of the endocervical samples also positive for *E. coli*, which is an interesting finding because previous studies have demonstrated the positivity of the *iha* gene in non-STEC uropathogenic *E. coli* (52). LEE-negative STEC strains have been found to be associated with clinical cases of HUS (53, 54). In the present work, we also studied the *sab* gene, which encodes an adhesin that promotes adherence to human epithelial cells, mediates the formation of biofilm, and is involved in intestinal colonization of LEE-negative STEC strains (15), but found no positive samples.

It is known that non-O157 STEC expressing Stx2 can affect the adult population, as observed in the outbreak that occurred in Germany in 2011, which produced approximately 3,500 cases of infection, 810 cases of HUS, and 39 deaths (55). Detailed studies of this outbreak demonstrated that the stx2-encoding phage can insert into the genome of non-O157 STEC strains and cause serious disease in the adult population, mostly women (56), although details on complications of pregnant vs non-pregnant women were not reported.

In summary, our results show the presence of a STEC O113:NT strain with Stx2a production in the endocervix of an asymptomatic woman during the first trimester of pregnancy. The patient (sample S14) received cephalexin, a broad-spectrum antibiotic, during the second trimester of gestation (20 weeks) to treat uterine inflammation and urinary infection. Probably antibiotic administration is the reason for no longer presence of the STEC strain in subsequent analysis of the endocervical samples during the second and third trimester as well as urine and fecal sample during the third trimester. At birth, the newborn presented a good general condition, and subsequent clinical checks of the mother and baby were normal. Therefore, the contributions of this study encourage us about the importance of the detection of *E. coli* during prenatal studies to prevent possible complications in pregnancy. Epidemiological studies related to the presence of vaginal STEC may establish its association with possible risks for the reproductive health of women.

## Data Availability Statement

The raw data supporting the conclusions of this article will be made available by the authors, without undue reservation.

## Ethics Statement

The studies involving human participants were reviewed and approved by Human Research Ethics Committee of the Prof. A. Posadas National Hospital, Buenos Aires, Argentina (Ref # 423 EMnPeSe/20). The patients/participants provided their written informed consent to participate in this study. Written informed consent was obtained from the individual(s) for the publication of any potentially identifiable images or data included in this article.

## Author Contributions

The authors contributions are as follows: Conceived and designed the experiments: SML, GN, CR, MA, SF, IC. Performed the experiments: SML, GN, SM, PN, MA, SF. Analyzed the data: SML, GN, SM, PN, MA, SF, IC. Contributed reagents/materials/analysis tools: SML, LP, PA, CR, MA, SF, IC. Wrote the paper: SML, GN, SF, IC. All authors contributed to the article and approved the submitted version.

## Conflict of Interest

The authors declare that the research was conducted in the absence of any commercial or financial relationships that could be construed as a potential conflict of interest.

## Publisher’s Note

All claims expressed in this article are solely those of the authors and do not necessarily represent those of their affiliated organizations, or those of the publisher, the editors and the reviewers. Any product that may be evaluated in this article, or claim that may be made by its manufacturer, is not guaranteed or endorsed by the publisher.
